# Preferences for and Experiences of an HIV-Prevention Mobile App Designed for Transmasculine People: Pilot Feasibility Trial and Qualitative Investigation

**DOI:** 10.2196/51055

**Published:** 2023-09-21

**Authors:** Jeb Jones, Gareth Butler, Meaghan Woody, Amanda D Castel, Paige Kulie, Martha Sheets, Ayden I Scheim, Sari L Reisner, Rachel Valencia, Minglun Wang, Joanne D Stekler, Patrick S Sullivan, Rob Stephenson

**Affiliations:** 1 Department of Epidemiology Emory University Atlanta, GA United States; 2 Department of Epidemiology George Washington University Washington, DC, DC United States; 3 Department of Epidemiology and Biostatistics Drexel University Philadelphia, PA United States; 4 Department of Epidemiology Harvard University Boston, MA United States; 5 Division of Allergy & Infectious Diseases University of Washington Seattle, WA United States; 6 Department of Systems Populations, and Leadership University of Michigan Ann Arbor, MI United States

**Keywords:** transmasculine, HIV, mHealth, preference, prevention, mobile app, transgender, STI, meta-analyses, app-based intervention, cisgender, sexual health, sexual risk behavior, smartphone

## Abstract

**Background:**

Transmasculine people are at risk for HIV; yet few HIV prevention interventions have been developed for this population. We adapted an existing HIV prevention smartphone app for cisgender men who have sex with men to meet the sexual health needs of transmasculine people.

**Objective:**

This study aims to assess the acceptability of the adapted app, Transpire, among transmasculine people living in Atlanta, Georgia, and Washington, DC, via in-depth interviews of participants in a pilot feasibility trial.

**Methods:**

Participants used the Transpire app for 3 months as part of a pilot study of the app. Eligible participants were aged 18-34 years. There were no eligibility criteria with respect to race and ethnicity, and most participants were non-Hispanic White. At the end of the follow-up, participants were invited to participate in web-based in-depth interviews to discuss their experiences using the app and feedback on design and content. Interviews were transcribed and coded using a constant comparative approach. Three main themes were identified: sexual behavior, app experiences and feedback, and pre-exposure prophylaxis.

**Results:**

Overall, participants found the app acceptable and thought that it would be a useful tool for themselves and their peers. Participants reported appreciating having a comprehensive information source available to them on their phones and reported learning more about HIV, sexually transmitted infections, and pre-exposure prophylaxis via the app. They also reported appreciating the inclusive language that was used throughout the app. Although the app included some resources on mental health and substance use, participants reported that they would have appreciated more resources and information in these areas as well as more comprehensive information about other health concerns, including hormone therapy. Representative quotes are presented for each of the identified themes.

**Conclusions:**

There is a desire to have greater access to reliable sexual health information among transmasculine people. Mobile apps like Transpire are an acceptable intervention to increase access to this information and other resources. More evidence is needed, however, from more racially and ethnically diverse samples of transmasculine people.

## Introduction

Transgender people in the United States are at increased risk for HIV and other sexually transmitted infections (STIs) compared to their cisgender counterparts [1]. Although transgender women account for most new HIV diagnoses among transgender people [2], transgender men and other gender-diverse people assigned female at birth who have sex with men also experience disproportionate HIV risk [3]. Transmasculine people, who are the focus of this study, are people who were assigned female sex at birth but whose current gender identity is masculine; thus, transmasculine is a broader group that is inclusive of transgender men. Meta-analyses have estimated the prevalence of HIV among transgender men and transmasculine people to be 3.2% [4] in the United States and 2.6% [5] worldwide, respectively. Transgender men accounted for approximately 6% of new HIV diagnoses among transgender and other gender-diverse people in the United States in 2020 [2].

Transmasculine people experience substantial barriers to accessing culturally competent care [6,7]. App-based interventions have been found to increase HIV testing among cisgender gay and bisexual men [8]. Similarly, mobile technology and telehealth platforms offer the potential to reduce barriers experienced by transmasculine people by, for example, providing accurate sexual health information and allowing people to access health care services remotely [9].

A key aspect of developing mobile health (mHealth)–based HIV and STI prevention interventions for transmasculine people is to understand the broader sexual health needs of this marginalized group. Patterns of sexual behavior, including heterogeneity of partner gender and type of sex engaged in, are more varied among transgender men compared to cisgender men [10], and transgender men need access to sexual health information that encompasses all of their needs. Many existing HIV and STI prevention smartphone apps have been developed to meet the needs of cisgender men who have sex with men (MSM) [11-13]. We adapted one such app, HealthMindr (Softura) [14], to meet the HIV prevention needs of transmasculine people [15].

Using a community-engaged approach, we created an adapted version of HealthMindr, rebranded as Transpire. Specifically, as described elsewhere [15], we created a community advisory board comprised of transmasculine people living in Atlanta, Georgia, and Washington, DC, to advise on rebranding the HealthMindr app, including the selection of the name Transpire, and on content to include in the app.

Similar to HealthMindr, Transpire contains information about pre-exposure prophylaxis (PrEP), HIV, and STIs; the ability to order HIV and STI self-test kits, condoms, and lube free of charge; and resources related to health insurance, substance use, and mental health. In addition, Transpire includes additional resources related to options for hormones and gender-affirming surgery, resources to identify trans-competent health care providers, and information on how to use internal and external condoms.

To assess the usability and acceptability of the Transpire app (Softura), we conducted a pilot study in which participants were able to use the app for 3 months, with access to all features including ordering HIV and STI self-test kits, condoms, and lube. Following completion of the trial, participants were invited to participate in in-depth interviews to discuss their experience with the app, recommendations for modifications, and experiences accessing HIV and STI prevention services. The goal of this study was to assess the experiences and recommendations of transmasculine research participants based on these interviews.

## Methods

### Participants

In total, 25 participants were recruited to participate in individual in-depth interviews from a pilot feasibility and acceptability study of Transpire, a smartphone app designed to meet the HIV prevention and sexual health needs of transmasculine people [15]. Recruitment into the pilot study was conducted via social media advertisements on Facebook and Instagram and flyers placed at community-based organizations that serve the trans community. Eligible participants were aged 18-34 years; identified on the transmasculine spectrum as transgender men, male, men, or another gender-diverse identity assigned female at birth; planned to remain in the study area (Atlanta and Washington, DC) for the duration of the trial; owned an Android or iOS smartphone; could read and understand English without assistance; reported having frontal/vaginal or anal sex with any partner in the past 12 months; and reported being HIV negative or never having been tested for HIV. All participants completed a self-administered web-based eligibility screener. Eligible participants completed a web-based informed consent process. Participants in the pilot study had access to the Transpire app for 3 months. After the 3 months, the participants were asked if they were interested in participating in an in-depth interview. Participants were compensated US $50 via electronic gift cards following the completion of the in-depth interview.

Recruitment occurred from November 2020 through October 2021; thus, this study overlapped with the early stages of the COVID-19 pandemic, including the period before vaccines became available. The sexual behavior and sexual health needs of participants during the pilot study were thus affected by social distancing to mitigate the spread of COVID-19.

### Structure of In-Depth Interviews

In-depth interviews were conducted over a Health Insurance Portability and Accountability Act (HIPAA)–compliant tool, Zoom (Zoom Video Communications Inc), by study staff (GB, MW, MS, and PK) trained by RS. Interviews were recorded and transcribed verbatim for analysis. Recordings were deleted following transcription. Each participant was asked the same set of interview questions using a semistructured interview guide with probing techniques when necessary. First, participants were asked about their history of HIV and STI testing prior to joining the study. Then, participants were asked about their overall experience with the Transpire app. This included likes, dislikes, most used functions of the app, most helpful functions of the app, and if the app was culturally competent on transgender topics and provided specific support to the transmasculine experience. After that, participants were asked a series of questions about their experiences with PrEP, whether they used the app’s PrEP Locator tool, and whether using the app affected their decision to start PrEP or receive care. Finally, participants were asked about their experience using Transpire to order HIV and STI test kits, condoms and other barrier prophylactics, and lube.

### Analysis

Transcripts were coded by 4 analysts using a constant comparative approach. Using this method, each coder first read 5 transcripts and created initial codes. Then, potential themes were discussed and finalized with consensus among the coders, along with the finalization of codes. Three major themes and 15 subthemes were created and used to code 25 transcripts. The 3 main themes and subthemes were: sexual behavior (health literacy, risk perception, access to care, external cues to action, and mental health), app experience and feedback (trans-competency/language, suggested app features, front-end app development, privacy, information comprehension, plans for future use, and ordering), and PrEP (misconceptions and accessibility).

### Ethics Approval

Participants provided informed consent over Zoom prior to the interview. All study procedures were reviewed and approved by the Emory University Institutional Review Board (protocol 00102006).

## Results

### Participant Demographics

A total of 25 in-depth interviews were conducted. The median age was 25 (IQR 21-29) years. Most participants identified as transgender male, transmasculine, or trans man (n=22, 88%) or gender diverse, gender nonbinary, or genderqueer (n=8, 32%; Table 1); participants were permitted to select all identities that applied, so these categories are not mutually exclusive. Most participants were non-Hispanic White (n=21, 84%) and had private health insurance (n=16, 64%). Approximately half of the participants were currently enrolled in school (n=13, 52%): 1 was enrolled in a general educational development program, 7 were enrolled in a 4-year college, and 5 were enrolled in graduate school.

**Table 1:** Demographic characteristics of in-depth interview participants (N=25).

Characteristic		Values
**Race or ethnicity**		
	Hispanic	1 (4)
	Non-Hispanic Black	1 (4)
	Non-Hispanic White	21 (84)
	Non-Hispanic other/multiracial	2 (8)
**Age (years), n (%)**		
	Median (IQR)	25 (21-29)
	18-24	10 (40)
	25-29	9 (36)
	30-34	6 (24)
**Gender identity, n (%)**		
	Transgender male, transmasculine, or trans man	22 (88)
	Gender diverse, gender nonbinary, or genderqueer	8 (32)
	Other	1 (4)
**Insurance, n (%)**		
	Private	16 (64)
	Public	5 (20)
	Other	0 (0)
	None	4 (16)
**Enrolled in school, n (%)**		
	Yes	13 (52)
	No	12 (48)
**Type of institution for those enrolled in school (n=13), n (%)**		
	General Educational Development program	1 (8)
	Four-year college	7 (54)
	Graduate school	5 (38)
**Highest level of education (n=12), n (%)**		
	High school diploma	1 (8)
	Some college, technical school, or vocational school	3 (25)
	Four-year college graduate	6 (50)
	Master degree or above	2 (17)
**Education level, n (%)**		
	High school diploma or less	2 (8)
	Some college technical school or vocational school	10 (40)
	College graduate and above	13 (52)
**Currently employed, n (%)**		
	Yes, full-time	10 (40)
	Yes, part-time	8 (32)
	No	7 (28)
**Annual income (US $), n (%)**		
	<$19,999	11 (44)
	$20,000-$39,999	8 (32)
	$40,000-$74,999	4 (16)
	>$75,000	2 (8)
	Prefer not to answer or do not know	0 (0)

### Sexual Behavior

#### Health Literacy

Most participants were already knowledgeable about sexual health prior to downloading the Transpire app; however, many reported that the app expanded their knowledge on certain sexual health topics such as PrEP, STI information, and HIV testing.

One participant described how much they appreciated having access to authoritative information about STIs.

It’s very hard to Google and be like, “Hey, what will this [infection] look like for me as a non-binary kid?” “Hey, what are the symptoms of this? What should I avoid, what should I not avoid?” And it was really nice having a comprehensive thing that I could just be like, “Oh, I don’t need to spend hours Googling it and stressing about it on WebMD.”

19 years old, non-Hispanic White

Another participant reported that the recommendations about the frequency of testing were helpful.

There was some information about how often you should get STI testing that I didn’t know, which I really should know, but I probably had been told before and forgot it, ‘cause that’s not something you really.... It’s not something you really internalize unless you’re actively having sex with multiple partners that it becomes part of your routine. So that was cool to have that all in one place.

21 years old, non-Hispanic White

#### Risk Perception

Many participants considered their HIV and STI risk to be low because they were in monogamous relationships or had reduced their sexual activity due to the COVID-19 pandemic.

One participant noted how their risk perception had been changed due to a recent STI diagnosis.

I became sexually active with multiple partners and last year I had chlamydia and I was like, “Okay, I should probably, like, make sure my bases are covered for the more serious stuff.”

25 years old, non-Hispanic White

When considering their risk in relation to PrEP initiation, another expected that their interest in HIV and STI prevention might increase as COVID-19 mitigation procedures and physical distancing decreased.

I’m like maybe when we’re not doing COVID stuff and...my risk factors are changing. [If] I’m having more sex and having sex with people that I don’t have full histories of, or I don’t have that, really developed, communicative relationship with, to do something like that. Like have that little preventative measure, done and knocked out.... Just routine. But yeah, I think it’ll be my landscape changing [that will] influence whether or not I start PrEP.

33 years old, non-Hispanic White

#### Access to Care

Most interviewees had taken an HIV test before, and most of the participants had been tested through their primary care physician or gynecologist. Some participants described barriers to accessing health services, such as finding the right provider and appointment availability.

When you’re looking for a safe space, like if it is like LGBTQ+ like centric, they might only have it at like let’s just say a window between like 12:00 to 6:00 on a Wednesday when some people might be working.... So sometimes when it is like a very like specific type of testing you wanna get just in terms of like the demographics and where you’re putting yourself, it’s just sometimes the logistical stuff or like there’s no parking available or like stuff like that.

26 years old, Non-Hispanic Asian

One participant described their negative experience with STI testing, which subsequently caused anxiety surrounding getting tested. Being able to order test kits through the app provided comfort in performing the STI tests at home.

The first experience was not good because I did end up having an STI, but it was a routine doctor’s appointment, so it was kind of a shock, and I was young. I think I was 17 when that happened. After that, it was kind of a follow-up preventative, maybe a year later. And since then, I’ve had a lot of anxiety surrounding getting tested or having those conversations with medical professionals, so I haven’t. Which has been irresponsible, but having access to something like this [the Transpire app], I feel like is helpful because I was able to order a kit through you guys, which I intend on sending back and following up on and having that extra measure of confidentiality and almost confidence in taking care of that side of my health. Very useful.

31 years old, non-Hispanic White

The same participant also felt that having at-home kits was empowering and easier than in-person testing.

But, I would say the ability now with technology and just being able to take healthcare into your own hands more, with testing kits and things like.... It makes it a lot easier to follow up and pursue taking care of my health.

31 years old, Non-Hispanic White

#### App-Implemented Behavior Change

Some participants felt that having access to the information in the app was enough to cause them to consider taking PrEP and getting tested more often.

Like I said, just going through everything, and re-reading everything, and then, like I said, reading about the PrEP, and the stuff that it’s supposed to be doing. And when they [the Transpire app] were asking about your sexual health, [chuckle] I’ll be honest, I think that it guilted me, because it was like, “Hey, when’s the last time you got tested for HIV?” And I was like, “It’s been a while.” And it was like, “How long has it been?” And I was like, “You know what, I don’t deal with guilting me very well, and guess what I’m gonna do now?”

29 years old, Hispanic

Yeah, definitely it made me think about testing more than I really ever had. I really had not considered getting HIV tested at all before, and then it just became very apparent that I should, so that was good, I think.

25 years old, Non-Hispanic White

In addition to having access to the app for 3 months, the monthly risk self-assessments completed by participants served as reminders to them to get tested, consider getting on PrEP, and keep their sexual health at the forefront of their mind.

Going through [the survey] asking about the questions about what you were doing and everything, and like doing check-ins and stuff, because it kinda helped me remember that I needed to start going to the doctor to get that stuff checked out again. So it was kind of really helpful to remind me.

29 years old, Hispanic

Only a few participants felt that the app influenced them to begin to take PrEP; most felt they were not engaged in activities that would put them at risk of acquiring HIV. Many participants thought the app was useful in that they knew they would be able to find a provider using the PrEP Locator tool if and when they wanted to begin PrEP.

Yeah. It definitely made me think about getting tested more frequently in the future because previously, I was like, oh, as long as I have sex with someone I trust, I don’t really need to get tested, blah, blah, blah, but seeing the app and seeing the concrete examples and the consequences of actions, I was like, okay, maybe scheduling tests every six months and talking with your partners about it could be more of a healthy thing.

19 years old, non-Hispanic White

I’m not sure ‘cause I feel like it might have been better suited if people felt that they were at risk to look more into it. But like I said, since I didn’t really see myself as being at risk, I didn’t seek out those sorts of follow ups.

25 years old, non-Hispanic White

#### Experiences of Stigma

One participant described their experiences with stigma in the health care setting, which led to preferences in who they seek HIV and STI care from.

I identified as a lesbian, so there was obviously where, especially, not necessarily where I lived, but with some of the providers stigma around being something other than straight and having sex obviously, and being Asian, and, you know, as time progressed, that made it uncomfortable to where it transitioned to where I got, and they asked my gender identity, and this was, you know, probably eight years ago and that’s like, “Oh man, now I’m gonna have to disclose this,” and then they’re like, “Oh, do you know you’re more at risk with HIV since you fall into a different gender.” And it just brings to that point of like the stigma and almost like I’m being judged by those provider and those people who were doing the testing.

31 years old, non-Hispanic other/multiracial

### App Experience and Feedback

#### Trans Competency and Language

Most participants agreed that the app had trans-competent and inclusive language and that the app felt like it was designed specifically for transmasculine people.

I feel like the language of the app was very friendly and it offered wellness information to people regardless of assigned sex at birth, I think, and what point of the transition they may or may not be in. And just using very neutral language was affirming, at least for me.

23 years old, non-Hispanic White

Another participant expressed approval of the straightforward language that was used to describe sexual behavior and sexual health needs, noting that sometimes people use euphemisms or language that is unclear or not relevant to their experience.

[The app used] language that felt relevant to my body and my partner’s body. And how we understand and engage with sex and like outside of the Cis gays and outside of either these strictly heterosexuals or even strictly gay context, that people like you understand sex to happen in. And just feeling like I got really specific information, right. I have this body, I have a cervix and I have a uterus that’s still doing uterus things and my partners have these things going on and we’re adding in these sorts of toys. So, what do we need to know?

33 years old, non-Hispanic White

One participant noted that they felt that transgender women and other gender-expansive people—beyond transmasculine people—could benefit from the Transpire app.

But I also think that it should be good for not just transmen, but I also think also transwomen, because I do know some of them do continue to still have anal sex. So I think it would still be useful for them, ‘cause I kind of.... Anybody who was going to do that, it kinda needs to be for that.

29 years old, Hispanic

#### Suggested App Features

Some interviewees noted that the Resources section would need to be updated as new information is released. Some also suggested having a chat function to talk to someone in real time for questions about PrEP and that they would like for the PrEP Locator tool [16] used in Transpire to include indicators of trans-competent providers in the results list. Another noted that they would have liked more comprehensive health information to be included:

Also, I know that it specifically is for sexual health and stuff like that but I think if it’s gonna cater to trans men, it could be really helpful to have more, I guess, in-depth information about how testosterone can impact it, just if you’re gonna include the resources anyways, you might as well throw in how physical changes and emotional changes that may not be related to sex, but still can be a really important factor.... Like I said, it’s really hard to find it online, so just having this little encyclopedia of trans information would be so cool.

18 years old, non-Hispanic White

Similarly, another participant felt that the app should have included more resources on mental health.

I feel like there is a focus on physical health, and I think that’s super important, obviously, and sexual health, obviously, but I think the mental, emotional stuff is also a big component when it comes to sexual health and trans spaces. Whether it be dysphoria, whether it be anything that’s not dysphoria. Yeah, I guess I was just expecting maybe some more mental health resources with it as well.

25 years old, non-Hispanic White

#### Functionality and Appearance

Most participants did not have strong feelings about the appearance and functionality of the app.

I liked that it was pretty user-friendly, so I didn’t have to navigate a difficult or complicated app. And yeah, it just felt like a good clean experience for an app, a health app.

23 years old, non-Hispanic White

Super easy to use. Super straight forward. I definitely, I clicked around on it a few times, I never had any issues with it, I will tell you that, it never froze up or I never had to go back to look for what I’m looking for, it’s very cut and clear. That’s nice.

31 years old, non-Hispanic White

#### Privacy

Most participants did not report privacy concerns and appreciated how orders from the app were shipped discreetly and did not require filing an insurance claim:

I like that it was discreet as well, if there wasn’t really, I didn’t feel like even though my name and my address and everything was there, I didn’t feel like anybody was able to find my information….

31 years old, non-Hispanic White

#### Information Comprehension

Many participants talked about the comprehensiveness of the app’s information sections and appreciated that a wide range of topics were covered at length.

Multiple participants described using the app’s information sources to learn more about PrEP in particular. Interviewees also discussed the added ease of having a centralized source of general information around various topics related to transmasculine health and well-being, as there are often unique barriers to accessing such resources for transmasculine people.

I learned a lot about PrEP. [chuckle] It wasn’t something that I had a whole lot of knowledge about previously… ‘Cause I don’t think I’d ever heard about like trans men or transmasc people being on PrEP before.

20 years old, non-Hispanic White

I found it pretty comprehensive, especially actually considering the state of sexual health resources for trans men out there, which is not too much or easily available.

22 years old, non-Hispanic White

Although a few participants explained they did not personally need some of the resources, such as legal aid or housing information, they described them as potentially helpful for other people in relevant situations.

I liked the vast amount of features. While I don’t necessarily need all of them. I think that it’s really good for people who need a little bit of extra help like navigating that situation.

22 years old, non-Hispanic White

#### Plans for Future Use

Many participants expressed that their current behaviors or circumstances did not put them in a position of needing some of the app’s features at the time; however, some expressed the intention of using resources or knowledge gained from the app in the future when they feel it is more relevant to their needs.

Yeah, pretty much that if I feel like I need more resources on sexual health and things, I would go there [Transpire app] first, for sure…. I didn’t use the app to actively search out information about PrEP yet. But if I am considering any sexual interaction with future partners, I will definitely use those resources.

23 years old, non-Hispanic White

So, I didn’t necessarily like use any of the resources, I just kind of checked out that area, and the reason why I didn’t really go in deeper was ‘cause I didn’t feel like I needed any resources at the time, but to know that it’s there and if I need it in future

31 years old, non-Hispanic White

#### Ordering

Participants provided feedback on the ordering home tests, condoms, and personal lubricant, and overall thought it was easy to use and included all the items they felt were necessary.

Found it pretty easy, just click on what the heck you want. There were a lot of different options, which is nice, ‘cause some people like different things. I was kinda like, “Ooh variety. Let’s see what this is about.” General ease of use, just general what’s available, so that’s what I liked about it.

22 years old, non-Hispanic White

I think it’s pretty comprehensive already, ‘cause it has the home STI testing kits and everything, it has a bunch of safe sex stuff, as well as just sex basics, like lube and everything. So I feel like that’s pretty comprehensive, honestly, for what you might need.

33 years old, non-Hispanic White

### PrEP

#### Misconceptions

Although the Transpire app included a variety of HIV and STI prevention resources, the focus of the app was on promoting PrEP as an HIV prevention tool. Thus, we specifically discussed PrEP knowledge and intentions with participants. Prior knowledge about PrEP varied, but most participants had previously heard of PrEP and had a general understanding of its purpose, although there were some misconceptions.

I know it was a pill that you took regularly to decrease your likelihood of contracting HIV. If as a person, who is HIV negative. And then, I also believe that PrEP is for people who are HIV positive and it’s supposed to lower their count to decrease the chances of passing it on.

31 years old, Asian

The app helped clear up prior misconceptions, such as the belief that PrEP was not approved for transmasculine people.

I didn’t honestly know that [PrEP] was approved in trans men, pretty much every doctor that I’ve talked to just implies that it’s for cis men, so it’s definitely helpful having that specified.

18 years old, non-Hispanic White

The information on there had me like ask my doctor about it, so definitely it made me actually consider it. Just because HIV is like a.... It’s still got that stigma of, “Oh, it’s only cis men who have sex with cis men.” You never... I guess I hadn’t really even thought about it before joining the study. It wasn’t something on my mind.

18 years old, non-Hispanic White

#### Accessibility

Overall, the main barrier to PrEP that was most frequently mentioned was concern about costs. Many people mentioned being uninsured or not knowing if their insurance covered PrEP.

I think for me, it’s the worries about whether or not insurance will cover it, things like that, so having the information about, if you don’t have insurance, what you can do on that [Transpire] app was really cool. For me, it’s definitely less of the, “Oh, I don’t wanna take it,” and more of the, “How am I gonna pay for it? Am I gonna get a doctor to prescribe it?” Things like that.

18 years old, non-Hispanic White

The first, the one that was the PrEP payment information that gave options on how to be able to pay for it, [laughter] ‘cause it’s expensive. And then when I looked at the provider, it did show, several places nearby that I could have gotten done. The best part that was there, like when you click on the provider thing, there’s a spot where you can actually click on PrEP for uninsured. And it narrowed everything down, which I thought was actually pretty cool.

29 years old, Hispanic

One participant expressed issues with finding a provider that is trans-competent and how that affects their access to care. This participant also noted that they hope to see a feature in the app to note trans competency in the clinics listed in the PrEP Locator.

Something that I noticed when.... I think it would be cool if there was a possibility.... I don’t know how this would work, but maybe something that allows people to sort of review PrEP providers because I noticed that, this is a personal or not a personal thing. This is a small thing, I notice when you go to the “find provider,” I’m pretty sure it shows one of the CVS’s [nearby] that has a reputation in the trans-masculine community indicator of being super transphobic.

31 years old, non-Hispanic other/multiracial

## Discussion

In this pilot study of an HIV prevention smartphone app, Transpire, for transmasculine adults, we found high acceptability of the app among participants. Participants appreciated that Transpire was a comprehensive source of information about HIV, STIs, and PrEP. Indeed, many participants commented that the app helped them to reconsider the importance of HIV and STI screenings. Participants commented that the individualized recommendations that the app provided based on their self-reported behavior helped them to recognize that more frequent HIV and STI screenings would be beneficial. Many appreciated the convenience of being able to order at-home HIV and STI self-tests through the Transpire app.

Although not many participants felt that PrEP was a good option for HIV prevention for themselves, many ascribed this to overall reduced sexual risk due to COVID-19 precautions and commented that they would be more likely to consider PrEP in the future because of the Transpire app. Data are limited on PrEP use among transmasculine individuals; however, in 1 sample, over half of trans-MSM participants had PrEP indications, half of whom had never used PrEP [17]. Thus, there is evidence of substantial unmet PrEP needs among transmasculine people. Several participants reported misconceptions about PrEP, and at least 1 thought that it was only for cisgender, not transgender, MSM. Even if they were not interested in starting PrEP during the study follow-up period, participants appreciated the comprehensive PrEP information included in the Transpire app, including information about how to pay for PrEP.

Participants noted several barriers to accessing sexual health care, including previous stigmatizing experiences, availability of appointments, and difficulty identifying trans-competent providers. Modifying existing resources like the PrEP Locator to be more useful for transgender and other gender-diverse users could have a meaningful impact on PrEP uptake among these populations.

With respect to suggestions to improve Transpire to better meet the needs of transmasculine people, a few themes emerged. There were recommendations to further refine the language in the app. At least 1 participant felt that the language used was more appropriate for cisgender men, a possible byproduct of the adaptation process of Transpire from HealthMindr, an app for cisgender MSM. Others felt that the language could be more inclusive of other trans identities, including trans women; however, the priority population for Transpire is transmasculine individuals (people assigned female at birth) given the dearth of research and interventions with this group. Another recommendation was to increase the mental health and gender dysphoria supports available through the app. Currently, Transpire includes brief screeners based on the Patient Health Questionnaire Screener [18] and a mental health and substance use treatment provider locator hosted by the Substance Abuse and Mental Health Services Administration [19]. Future versions of Transpire could incorporate more robust resources to meet the needs of transmasculine users. We are not aware of any standalone interventions that might be incorporated into the app; however, additional information about available treatment options for mental health conditions and substance use problems, including links to external resources, could easily be incorporated. Overall, there were no privacy concerns reported, and participants felt comfortable using the app and ordering sexual health care supplies via the app.

Other smartphone apps based on the HealthMindr parent app have been shown to be effective tools for increasing the uptake of HIV prevention services among cisgender MSM [8], and the results of this study indicate that transmasculine participants found a HealthMindr-based HIV prevention app to be acceptable to use and a potentially valuable tool to increase knowledge about sexual health needs and provide access to health care resources. In addition to the HIV prevention resources included in Transpire, participants expressed a desire for a smartphone app that included information and resources on a broad array of health topics. For many participants, HIV was not a primary health concern, so including additional health topics might broaden the appeal of Transpire or similar apps for transmasculine people.

This study has several limitations. Most participants were non-Hispanic White, were currently enrolled students, were in postsecondary programs or had a college education or higher, had private health insurance, and were recruited from 2 urban settings in the southern region of the United States. This reflects the demographics of the study population in the broader source study, of whom 77% were non-Hispanic White, 10% were Hispanic, and 7% were non-Hispanic Black [15]. Thus, these results are not generalizable to the broader transmasculine population; it will be important to replicate these procedures with a more racially and ethnically diverse population. The eligibility criteria for the overall study were also very broad with respect to sexual risk behavior, thus our study population did not necessarily represent those transmasculine individuals at highest risk of HIV transmission. Participants were also only able to use the app for a period of 3 months during a time in which many reported reducing their sexual behavior due to the COVID-19 pandemic; thus, many participants did not have an opportunity to use the app during a time in which its features might have been more useful.

We found high levels of interest in an HIV prevention app for transmasculine people. Participants also made a number of suggestions to improve the app and make it more relevant to the health care needs of transmasculine people. Smartphone apps have the potential to improve both sexual and overall health of transmasculine people by providing accurate health-related information, linking users to health care providers, and allowing users to order tools, such as self-test kits, that empower them to conduct screenings in privacy of their home. The findings highlight the importance of tailored interventions designed to meet the sexual health needs of transmasculine individuals.

The adaptation of the Transpire app from HealthMindr was conducted through a community-engaged process, which was a key factor in developing an app that addressed the needs of transmasculine people. Continuing engagement with potential end users and other stakeholders in the community will be key to developing an app that will have the greatest impact on the health of transmasculine people.

## Abbreviations

HIPAA: Health Insurance Portability and Accountability Act
mHealth: mobile health
MSM: men who have sex with men
PrEP: pre-exposure prophylaxis
STI: sexually transmitted infection

## Data Availability

Data from this study are available upon request from the first author.
